# Insight into the Local Surface Plasmon Resonance Effect of Pt-SnS_2_ Nanosheets in Tetracycline Photodegradation

**DOI:** 10.3390/molecules29225423

**Published:** 2024-11-17

**Authors:** Mao Feng, Tianhao Zhou, Jiaxin Li, Mengqing Cao, Jing Cheng, Danyang Li, Jian Qi, Feifei You

**Affiliations:** 1 Textile School, Zhejiang Fashion Institute of Technology, Ningbo 315211, China; xgf@msn.com; 2 College of Textile and Clothing, Yancheng Institute of Technology, Yancheng 224051, China; 17311504196@163.com (T.Z.); 19705102579@163.com (J.L.); 18164327219@163.com (M.C.); 19705100129@163.com (J.C.); 3 Sichuan Provincial Engineering Research Center of Functional Development and Application of High Performance Special Textile Materials, Chengdu Textile College, Chengdu 611731, China; 4 State Key Laboratory of Biochemical Engineering, Institute of Process Engineering, Chinese Academy of Sciences, Beijing 100190, China; 5 School of Chemical Engineering, University of Chinese Academy of Sciences, Beijing 100049, China

**Keywords:** sheet structure, Pt/SnS_2_, tetracycline, photodegradation, heterogeneous junction

## Abstract

Constructing highly efficient catalysts for the degradation of organic pollutants driven by solar light in aquatic environments is a promising and green strategy. In this study, a novel hexagonal sheet-like Pt/SnS_2_ heterojunction photocatalyst is successfully designed and fabricated using a hydrothermal method and photodeposition process for photocatalytic tetracycline (TC) degradation. The optimal Pt/SnS_2_ hybrid behaves with excellent photocatalytic performance, with a degradation efficiency of 91.27% after 120 min, a reaction rate constant of 0.0187 min^−1^, and durability, which can be attributed to (i) the formation of a metal/semiconductor interface field caused by loading Pt nanoparticles (NPs) on the surface of SnS_2_, facilitating the separation of photo-induced charge carriers; (ii) the local surface plasmon resonance (LSPR) effect of Pt NPs, extending the light absorption range; and (iii) the sheet-like structure of SnS_2_, which can shorten the transmission distance of charge carriers, thereby allowing more electrons (*e^−^*) and holes (*h^+^*) to transfer to the surface of the catalyst. This work provides new insights with the utilization of sheet-like structured materials for highly active photocatalytic TC degradation in wastewater treatment and environmental remediation.

## 1. Introduction

Antibiotics are crucial for preventing and treating bacterial infections in humans and infectious diseases in livestock [1,2]. Tetracycline (TC), as a broad-spectrum antibiotic, is renowned for its potent antibacterial properties and better cost-effectiveness [3,4]. Unfortunately, due to the chemical stability and resistance to biodegradation of TC, the residual TC will eventually be released into the soil and aquatic environments, causing serious pollution to the environment, and then posing significant threats to ecosystems and human health [5,6]. Despite various wastewater treatment methods such as adsorption, biological treatment, membrane separation, and advanced oxidation processes, the drawbacks of secondary pollution, high energy consumption, low efficiency, and complicated treatment processes restrict the widespread application of these strategies [7,8]. Therefore, developing effective, environmentally friendly, and economical strategies to remove TC antibiotic residues in aquatic environments is an important and urgent issue of crucial significance to the ecological environment, people’s health, and the achievement of sustainable development [9].

So far, photocatalytic TC degradation driven by inexhaustible green solar energy is considered one of the most promising solutions owing to its high efficiency and stability, low toxicity and cost, sustainability, and recyclability [10,11,12]. In a nutshell, photocatalysts generate photo-generated *e^−^*/*h^+^* pairs with redox properties under light illumination, which can transfer to the surface of the photocatalyst and react with H_2_O and O_2_ to produce active species such as hydroxyl radicals (·OH) and superoxide anion radicals (·O_2_^−^), and then the radicals are exploited to degrade TC to form harmless products [13,14]. The key factors enhancing the photodegradation efficiency include increasing light capture and absorption, hindering the fast recombination of photo-induced charge carriers, and improving mass transport. Thus, the delicate design of the macro-structure and elaborate choice of micro-composition are highly required to achieve satisfactory degradation efficiency.

Two-dimensional (2D) sheet-like materials possess unique merits in the field of photocatalysis [15,16,17,18,19,20,21,22,23,24,25], such as shortening the transport path of charge carriers to make more photo-excited *e^−^* and *h^+^* pairs to participate in reactions on the surfaces of the catalysts, exposing specific crystal planes to provide more reactive sites, and preventing catalysts from aggregating together during the reaction process [26,27,28,29,30,31,32,33,34,35,36,37]. Additionally, the semiconductors with 2D structures, such as TiO_2_ [38], g−C_3_N_4_ [39], Fe_2_O_3_ [40], ZnO [41], and SnS_2_ [42], etc., are considered to be promising candidates for the efficient photocatalytic degradation of TC. Among them, SnS_2_, an n-type semiconductor with a fascinating band gap of ~2.2 eV and the advantages of being non-toxic, harmless, easy to prepare, inexpensive, and environmentally friendly, has been extensively studied for the photodegradation of water pollutants [43,44]. However, pure SnS_2_ tends to suffer from the weakness of rapid carrier recombination, restricting its photocatalytic efficiency. In view of this, constructing heterojunctions as well as decorating noble metal NPs is an effective method to overcome this intrinsic limitation. Noble metals, such as Ag, Au, Pt, and Pd, have been verified to display unique LSPR effects and are extensively employed as co-catalysts to enhance photocatalytic efficiency [45]. The noble metal NPs can absorb and scatter visible light, causing a strong local electromagnetic field, which benefits the excitation, separation, and transfer of photo-induced carriers, improving the photocatalytic activity. Vishal et al. [46] successfully designed and synthesized a novel ternary Z-Scheme Ag/HAp/SnS_2_ catalyst for the photodegradation of metronidazole, which behaved with excellent photodegradation efficiency because of the formation of heterogeneous junctions and Ag NPs acting as a charge transfer medium and *e^−^* accumulators delaying *e^−^*/*h^+^* recombination. Li et al. [47] successfully prepared a hollow-structured Pt/TiO_2_ hybrid as a catalyst for photocatalytic TC degradation, exhibiting great photodegradation performance and durability attributed to the formation of Schottky junctions and the LSPR effect of Pt.

In this work, Pt NPs loaded on sheet-like-structured SnS_2_ hybrids were designed and synthesized through a simple hydrothermal process and photodeposition reaction toward TC photodegradation. Pt NPs extended the light absorption range due to the LSPR effect as well as captured the *e^−^* of SnS_2_ with plasmonic hot *h^+^* caused by tough electron oscillation of LSPR excitation. Additionally, Pt NPs also played the role of *e^−^* grooves, which could promote the separation of charge carriers and enrich *e^−^*. Benefiting from the Schottky junction constructed between Pt and SnS_2_ and the natural advantages of the sheet-like structure of a short carrier transfer path and more exposed active reaction sites, the optimal specimen showed outstanding photocatalytic TC degradation activity with a degradation efficiency of 91.27% under light illumination for 120 min, a rate constant of 0.0194 min^−1^, and durability in five cycles without apparent activity reduction. Thus, we believe that the sheet-like-structured Pt/SnS_2_ heterogeneous junction catalyst provides a different strategy for the construction of highly efficient photocatalysts for the degradation of water pollutants.

## 2. Results and Discussion

### 2.1. Morphological and Structural Characterization

**Figure 1:**
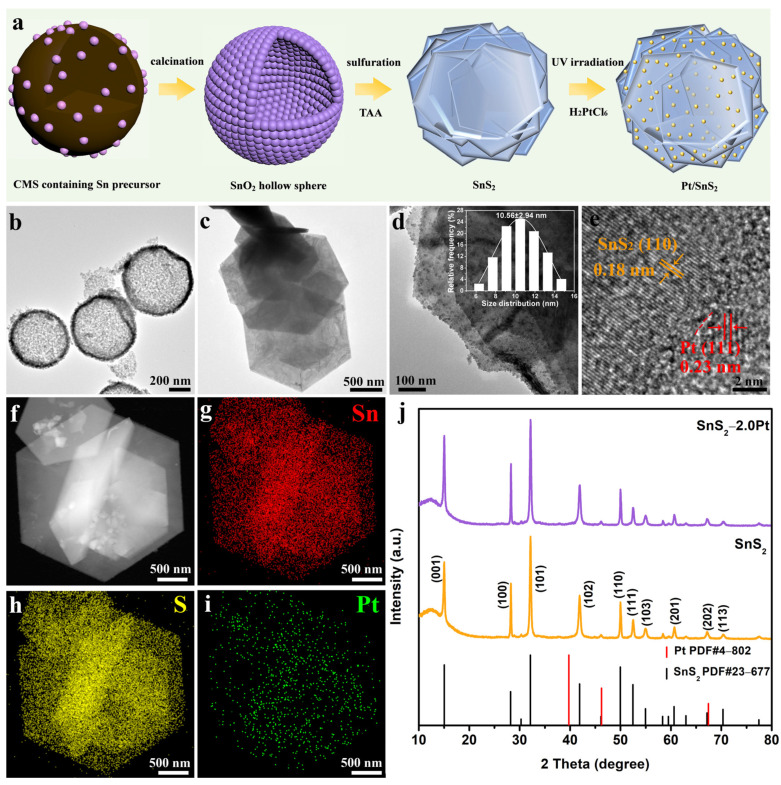
(**a**) Schematic illustration of the synthesis process of sheet-like-structured SnS_2_ and Pt/SnS_2_ hybrids; TEM images of (**b**) SnO_2_ hollow sphere, (**c**) SnS_2_ and (**d**) SnS_2_-2.0Pt with inset of particle size distribution of Pt NPs; HRTEM image of (**e**) SnS_2_-2.0Pt; (**f**) HAADF−STEM and (**g**–**i**) elemental distribution images of SnS_2_−2.0Pt; and (**j**) XRD patterns of SnS_2_ and SnS_2_−2.0Pt with standard diffraction peaks of SnS_2_ and Pt (vertical lines).

The synthesis procedure of a sheet-like Pt/SnS_2_ hybrid is illustrated in Figure 1a. Briefly, the hard template method was used to synthesize the SnO_2_ hollow sphere, with CMS and SnCl_4_ as a sacrificial template and metal ion precursor, and then sheet-like-structured SnS_2_ was obtained through sulfuration treatment in the presence of TAA. After that, 2D heterogeneous junction Pt/SnS_2_ hybrids were prepared via a subsequent photodeposition process. As shown in the transmission electron microscopy (TEM) image (Figure 1b), the SnO_2_ hollow spheres display a coarse surface with an outer diameter of 600–700 nm. After sulfuration, it could be obviously observed that the SnO_2_ hollow sphere became the hexagonal sheet-like-structured SnS_2_ (Figure 1c). It must be pointed out that the structure has changed from three-dimensional (3D) to two-dimensional (2D), resulting in a significant increase in size. Figure 1d demonstrates the TEM of the Pt/SnS_2_ hybrid, and the Pt NPs with an average diameter of 10.56 nm are uniformly dispersed on the surface of the SnS_2_ sheet. The high-resolution TEM (HRTEM) image of the Pt/SnS_2_ hybrid displayed in Figure 1e indicates that the heterojunction formed by SnS_2_ and Pt and the Pt NPs are tightly anchored at the surfaces of SnS_2_. Two lattice fringes were measured with the interplanar distances of 0.18 and 0.23 nm, which corresponded to the (110) plane of SnS_2_ [48] and the (111) plane of Pt [49], respectively. Furthermore, the corresponding high-angle annular dark-field scanning transmission electron microscopy (HAADF−STEM) and elemental mapping images (Figure 1f–i) of the Pt/SnS_2_ hybrid verified that the elements of Sn, S, and Pt were well dispersed throughout the catalyst and further confirmed the uniform distribution of Pt nanoparticles loaded on the surfaces of sheet-like Pt/SnS_2_, indicating the formation of ample intimate heterointerfaces between Pt and SnS_2_.

The crystallographic properties and phase composition of the SnS_2_ and SnS_2_−2.0Pt hybrids were investigated by X-ray diffraction (XRD) patterns (Figure 1j). The typical diffraction spectrum with specific peaks of pure SnS_2_ is indexed by hexagonal SnS_2_ (PDF#23-0677) [48], and the diffraction peaks located at 15.029, 28.199, 32.124, 41.886, 49.960, 52.451, 54.960, 60.619, 67.152, and 70.333° are well attributed to (001), (100), (101), (102), (110), (111), (103), (201), (202), and (113) crystal facets with lattice constants of a = b = 3.6486 Å and c = 5.8992 Å. The absence of no impurity peaks demonstrates that pure SnS_2_ has been successfully fabricated. As for SnS_2_−2.0Pt, loading Pt NPs does not influence the crystalline structure of SnS_2_. The deposition of Pt NPs at SnS_2_−2.0Pt concentrations was not detected in the XRD pattern with JCPDS card no. 4−802 because of the small particle size of Pt NPs with highly uniform dispersion onto the sheet-like SnS_2_. Nevertheless, the energy-dispersive spectroscopy of SnS_2_−2.0Pt illustrated in Figure S1 displays that Pt NPs do exist.

### 2.2. XPS Analysis

**Figure 2:**
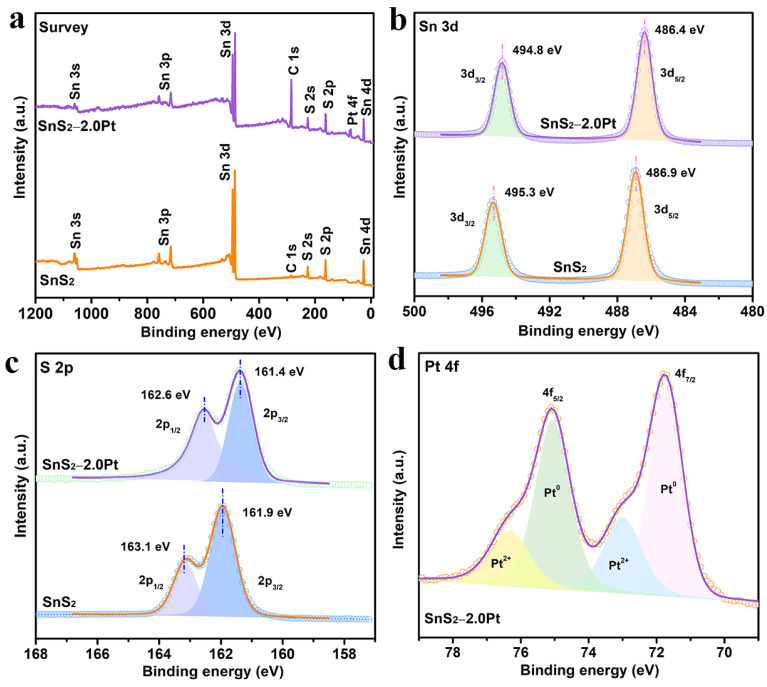
XPS spectra of SnS_2_ and SnS_2_−2.0Pt: (**a**) survey; high-resolution XPS spectra of (**b**) Sn 3d, (**c**) S 2p, and (**d**) Pt 4f.

An X-ray photoelectron spectroscopy (XPS) test was used to investigate the element composition and valence state of as-prepared samples. Figure 2a depicts the survey XPS spectra of SnS_2_ and SnS_2_−2.0Pt, and the peaks of Pt only can be observed in SnS_2_−2.0Pt, proving once again the successful modification of Pt onto SnS_2_. The peaks at 486.9 and 495.3 eV in Figure 2b mainly focus on Sn 3d_5/2_ and Sn 3d_3/2_, belonging to the binding energies of Sn^4+^ states [50]. And the high-resolution XPS scans of the S 2p spectrum exhibit two peaks with the binding energies of 161.9 and 163.1 eV (Figure 2c), corresponding to S 2p_3/2_ and S 2p_1/2_, respectively, confirming the chemical state of S with −2 valence in the SnS_2_ sheet. However, compared to the pure SnS_2_, the binding energies of S 2p and Sn 3d for SnS_2_−2.0Pt showed a slightly negative shift toward a lower direction of 0.5 eV (S 2p) and 0.5 eV (Sn 3d), indirectly confirming the closed interaction between Pt NPs and SnS_2_, which shows that the *e^−^* of SnS_2_ migrate to Pt at the interface. As shown in Figure 2d, the Pt 4f XPS spectrum of SnS_2_−2.0Pt can be divided into two double peaks. The peaks located at 71.7 eV and 75.1 eV belong to the Pt 4f_7/2_ and Pt 4f_5/2_ of metal Pt^0^, while the peaks at 73.0 eV and 76.3 eV correspond to the Pt 4f_7/2_ and Pt 4f_5/2_ of Pt^2+^ [51,52,53,54], existing in the interfaces of Pt and SnS_2_ or the oxidized Pt atoms [55]. Moreover, the ratio of Pt^0^/Pt is 70.18% (Table S1), demonstrating that Pt is mainly presented in the metallic form.

### 2.3. Photocatalytic TC Degradation Evolution

**Figure 3:**
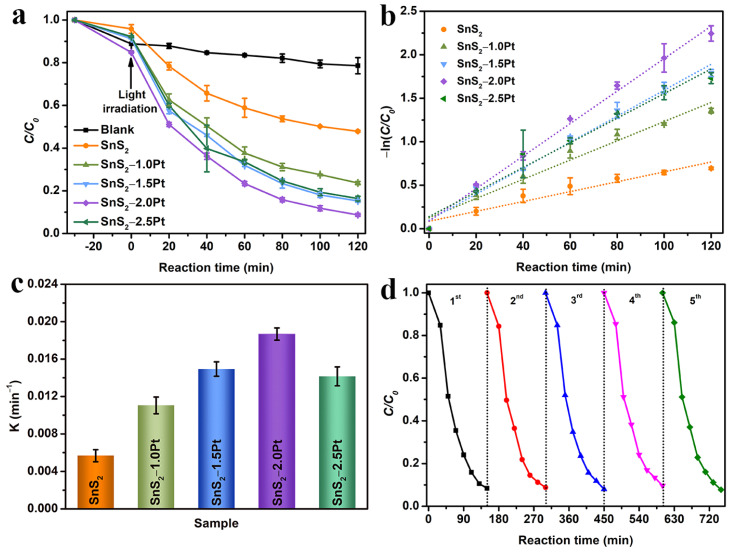
(**a**) Photocatalytic activities of all as-prepared samples during the degradation of TC, (**b**) kinetic curves, (**c**) the reaction rate constant, and (**d**) durability tests of SnS_2_−2.0Pt.

The photocatalytic property tests of TC degradation for all as-prepared samples were carried out under 300 W Xe lamp irradiation. The standard curve of absorbance vs. varied concentrations of TC is displayed in Figure S2. The initial TC solution containing catalysts was stirred for 30 min in a dark environment, aiming to achieve adsorption/desorption equilibrium between TC and the catalyst before illumination. Firstly, the blank experiment was conducted. The test result (Figure 3a) showed that the TC was difficult to degrade under the condition of no light irradiation, while the concentration of TC significantly decreased in the existence of light and the catalyst. The pure SnS_2_ demonstrated a degradation efficiency of 52.15% for TC within 120 min. And for Pt/SnS_2_ hybrids, there was a rise in the Pt NP amount loaded on the SnS_2_ sheet. The degradation activity was improved; however, the excess loading amount of Pt NPs caused a decrease in the performance of TC degradation. The degradation efficiency of TC was 76.32%, 84.76%, 91.27%, and 83.65% for SnS_2_−1.0Pt, −1.5Pt, −2.0Pt, and −2.5Pt, respectively, and SnS_2_−2.0Pt showed optimal photocatalytic TC degradation performance behaviors.

Then, the reaction kinetics of the catalytic process for all as-prepared samples was studied using the first-order reaction kinetic equation of −ln(*C_t_*/*C_0_*) = *kt*, where *t* is reaction time, *C_t_* stands for the concentration of TC after t min light irradiation, *C_0_* is the concentration of TC after adsorption/desorption equilibrium in a dark environment, and *k* is the reaction rate constant (min^−1^). The fitting data (Figure 3b) demonstrate a very good linear relationship between −ln(*C_t_*/*C_0_*) and *t*, and the *R*^2^ values of SnS_2_, SnS_2_−1.0Pt, −1.5Pt, −2.0Pt, and −2.5Pt are 0.9301, 0.9619, 0.9845, 0.9927, and 0.9699. And the slopes of the fitted lines are represented by the value of *k*, which is 0.0057, 0.0111, 0.0149, 0.0187, and 0.0142 min^−1^ for SnS_2_, SnS_2_−1.0Pt, −1.5Pt, −2.0Pt, and −2.5Pt (Figure 3c). Notably, SnS_2_−2.0Pt demonstrates the best performance of TC photodegradation among all samples and also possesses strong competitiveness compared with the published papers with the SnS_2_-based materials under similar reaction conditions [48,56,57,58,59,60] (Table 1). In addition, Figure 3d shows the recyclability and stability of SnS_2_−2.0Pt, in which the degradation rate of TC has no obvious change during five cycles, suggesting the stability of SnS_2_−2.0Pt for photocatalytic TC degradation. And the high stability of the SnS_2_-2.0Pt sample crystal structure and morphology can also be proved by the XRD pattern and TEM image (Figure S3). Furthermore, the pH value of the initial TC solution as a major effect parameter was studied during the process of TC degradation. As shown in Figure S4, SnS_2_−2.0Pt demonstrated excellent performance under a wide pH range with a TC degradation efficiency of 92.50%, 91.56%, 88.17%, and 83.28 under the initial solution pH values of 3, 5, 9, and 11, respectively. The degradation rate decreased slightly but not significantly in an alkaline environment, indicating that SnS_2_−2.0Pt could exhibit good performance in a wide range of pH values.

### 2.4. Photoelectronic Tests

**Figure 4:**
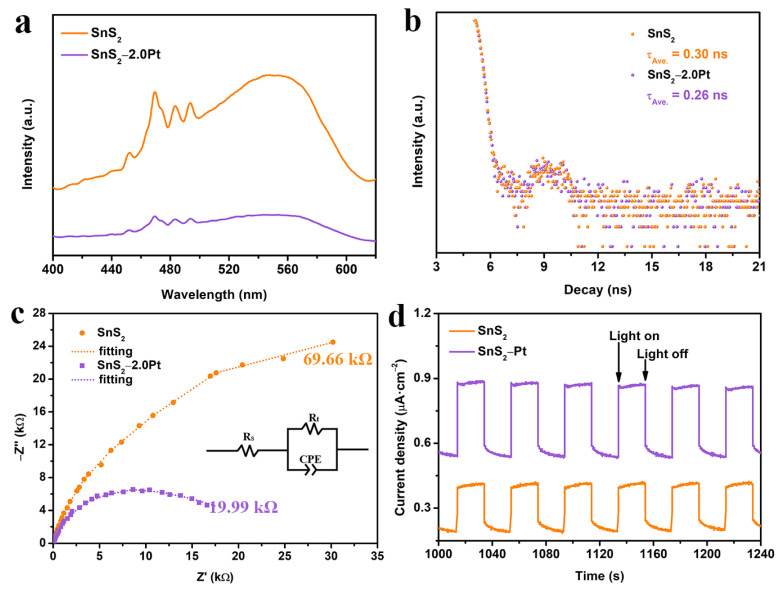
Photoelectronic characterizations of SnS_2_ and SnS_2_−2.0Pt: (**a**) steady-state PL spectra, (**b**) time-resolved PL spectra, (**c**) EIS Nyquist plots and the fitting circuit diagram (inset), and (**d**) photocurrent density−time curves.

In order to investigate the reasons why the SnS_2_−2.0Pt sample behaved with such properties during the photodegradation of TC, a series of photoelectronic characterizations were carried out. Figure 4a shows the steady-state photoluminescence (PL) spectra of SnS_2_ and SnS_2_−2.0Pt with the peak position located at ~550 nm. And SnS_2_−2.0Pt displays a weaker peak intensity compared to that of pure SnS_2_, suggesting that the heterogeneous structures constructed between SnS_2_ and Pt NPs hinder the recombination of photo-induced *e^−^*/*h^+^* pairs [61]. In addition, time-resolved PL was used to study the characteristics of photo-excited charge carriers. As illustrated in Figure 4b, the average lifetimes (τ_Ave._) of SnS_2_ and SnS_2_−2.0Pt were calculated to be 0.30 and 0.26 ns using the biexponential function, respectively. The shorter average fluorescence lifetime of SnS_2_−2.0Pt demonstrated the improved transfer and separation efficiency of charge carriers [62]. Moreover, the electrochemical impedance spectra (EIS) exhibited in Figure 4c show the fitted semicircle diameter values of 69.66 and 19.99 kΩ for SnS_2_ and SnS_2_−2.0Pt, respectively, and smaller semicircles of SnS_2_−2.0Pt indicate lower charge carrier transfer resistance. Furthermore, SnS_2_−2.0Pt shows higher photocurrent density than that of SnS_2_ (Figure 4d), demonstrating a promotion of the generation and separation efficiency of charge carriers due to the heterojunctions in SnS_2_−2.0Pt. All these characterization results confirm that the generation, separation, and transfer of photo-induced *e^−^*/*h^+^* pairs can be enhanced in SnS_2_−2.0Pt during photocatalytic TC degradation [63,64], resulting in higher photocatalytic activity.

### 2.5. Mechanism Analysis

**Figure 5:**
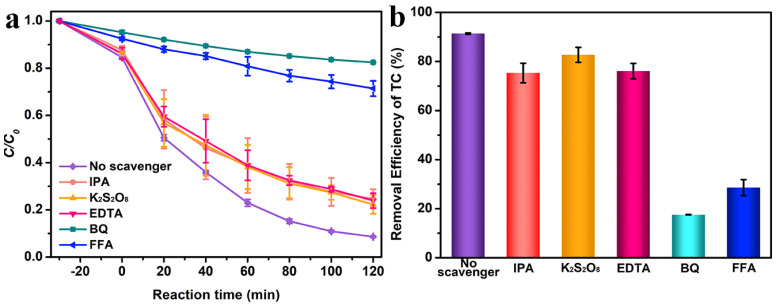
(**a**) Photocatalytic activities of the degradation of TC and (**b**) TC removal efficiency with the SnS_2_−2.0Pt catalyst in the presence of various scavengers.

Free-radical trapping experiments were implemented to examine the main active species in TC photodegradation. Normally, the active substances, *e^−^*, hydroxyl radicals (∙OH), *h^+^*, superoxide radicals (∙O_2_*^−^*), and singlet oxygen (^1^O_2_), were generated during the process of photocatalytic TC degradation, which could be captured using the scavengers of IPA, K_2_S_2_O_8_, EDTA, BQ, and FFA, respectively. As displayed in Figure 5a,b, the TC degradation efficiency decreased after adding IPA, K_2_S_2_O_8_, EDTA, BQ, and FFA, with the degradation efficiency of 75.30%, 82.72%, 76.11%, 17.56%, and 28.60%, respectively. BQ was a great obstacle to TC degradation, followed by FFA, and IPA, K_2_S_2_O_8_, and EDTA illustrated a relatively small impact on TC degradation. Thus, ∙O_2_*^−^* played a dominant role in the degradation process of TC, followed by ^1^O_2_, and *e^−^*, ∙OH, and *h^+^* played an auxiliary role [65,66].

**Figure 6:**
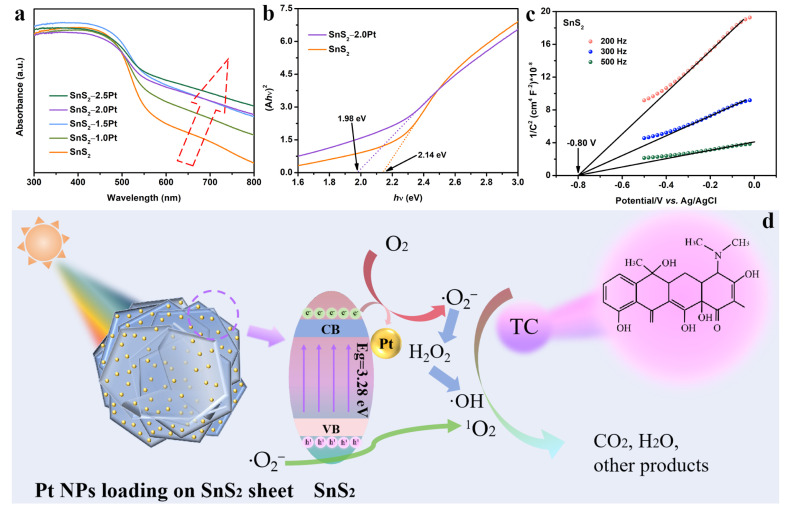
(a) UV−Vis spectra of all samples, (**b**) Tuac curves of SnS_2_ and SnS_2_−2.0Pt, (**c**) Mott−Schottky plots of SnS_2_ and (**d**) the main proposed photocatalytic TC degradation mechanism diagram of the sheet-like SnS_2_−2.0Pt heterogeneous catalyst.

To unveil the catalytic reaction mechanism, corresponding tests were conducted to define the energetic band structure. The UV−Vis light absorption spectra of SnS_2_ and SnS_2_−1.0Pt, −1.5Pt, −2.0Pt, and −2.5Pt are revealed in Figure 6a. The light absorption edge is approximately 580 nm for pure SnS_2_. After loading Pt NPs on SnS_2_, the light absorption of Pt/SnS_2_ hybrids is significantly enhanced compared to that of pure SnS_2_, which contributes to the LSPR effect of Pt. The Tauc plots can be obtained through the UV−Vis light absorption data. And then the energy band gap (*E_g_*) of as-prepared samples can be determined according to the intercept of the straight lines of the Tauc curves on the *x*-axis. As exhibited in Figure 6b, the *E_g_* values of SnS_2_ and SnS_2_−2.0Pt are 2.14 and 1.98 eV, respectively. In addition, the Mott−Schottky measurement is used to confirm the location of the flat band (*E_f_*). Figure 6c demonstrates the Mott−Schottky plots under various frequencies of SnS_2_, whose slopes are positive, indicating an n-type semiconductor of SnS_2_. As for the n-type semiconductor, compared to the position *E_f_*, the conduction band (*E_CB_*) potential is negative 0.1 V [67]. According to the intercept of the straight section of the Mott−Schottky curves with various frequencies on the *x*-axis, the *E_f_* potential of SnS_2_ is −0.60 V (vs. NHE). And the position of the *E_CB_* of SnS_2_ is −0.70 V (vs. NHE). The valence band potential (*E_VB_*) of SnS_2_ is calculated to be 1.44 V (vs. NHE) from the following formula: *E_VB_* = *E_g_* + *E_CB_* [68]. Thus, the proposed main mechanism of TC photodegradation for the Pt/SnS_2_ hybrid is illustrated (Figure 6d) and the related reactions are as follows: Under light irradiation, the *e^−^* are excited and move from the valence band (VB) to the conduction band (CB) of SnS_2_; in the meantime, an equal quantity of *h^+^* is produced in the VB of SnS_2_ (Reaction 1). Because of the tough electron oscillation induced by LSPR excitation, the generated plasmonic hot *h^+^* can capture the *e^−^* in the CB of SnS_2_, which can effectively suppress the recombination of photo-excited *e^−^*/*h^+^* pairs. Then, the oxygen is reduced to ∙O_2_*^−^* (Reaction 2) by the *e^−^*. Subsequently, the ^1^O_2_ can be generated through ∙O_2_*^−^* reacting with *h^+^* (Reaction 3). Finally, TC is degraded by the active species (Reaction 4).

Pt/SnS_2_ + light → *e*^−^ + *h^+^* (1)

O_2_ + e^−^ → ∙O_2_^−^ (−0.33 V vs. NHE) (2)

∙O_2_^−^ + h^+^ → ^1^O_2_ (0.67 V vs. NHE) (3)

∙O_2_^−^/^1^O_2_ + TC → CO_2_ + H_2_O + other products (4)

## 3. Materials and Methods

### 3.1. Materials

Thioacetamide (TAA) and sodium sulfate anhydrous (Na_2_SO_4_) were purchased from Shanghai Aladdin Biochemical Technology CO., Ltd., Shanghai, China. Tin (IV) chloride pentahydrate (SnCl_4_·5H_2_O), chloroplatinic acid (H_2_PtCl_6_), absolute ethanol, methyl alcohol, isopropyl alcohol (IPA), TC, potassium persulfate (K_2_S_2_O_8_), ethylene diamine tetraacetate dehydrate (EDTA), furfuryl alcohol (FFA) and p-ben-zoquinone (BQ) were purchased from Shanghai Macklin Biochemical Technology CO., Ltd., Shanghai, China. Sucrose was supplied from Xilong Science Co., Ltd., Shantou, China. All the above chemicals were analytical reagent grade and were utilized directly without further purification.

### 3.2. Preparation of Photocatalysts

#### 3.2.1. Preparation of Carbonaceous Microsphere (CMS) Templates

The details can be seen in Supporting Information.

#### 3.2.2. Preparation of SnO_2_ Hollow-Structured Microspheres

The synthesis process can be found in Supporting Information.

#### 3.2.3. Preparation of Sheet-Like SnS_2_

Firstly, 1.0 g TAA and 100.0 mg SnO_2_ hollow spheres were gradually added into 30.0 mL deionized (DI) water, and the suspension was vigorously stirred with a magnetic stirrer for 30 min at room temperature to form a mixed homogeneous solution. Next, the obtained suspension was placed in a 50 mL stainless steel autoclave and crystallized in a 180 °C oven for 3 days. After the stainless steel autoclave was naturally cooled to room temperature, the product was separated by centrifugation and washed several times with deionized water and ethanol in sequence. Finally, the product was dried in a 60 °C oven for 24 h to obtain the yellow powder.

#### 3.2.4. Synthesis of Pt/SnS_2_ Hybrids

Firstly, 2 mg/mL H_2_PtCl_6_ aqueous solution was prepared. Subsequently, 40 mL of deionized water, 10 mL of methanol, and different volumes of H_2_PtCl_6_ solutions of 1.05, 1.57, 2.10, and 2.62 mL were poured into the beakers in sequence, and this was stirred for 30 min to form a uniform solution. Then, 100 mg of SnS_2_ (Pt/SnS_2_ mass ratios were 1.00, 1.50, 2.00, and 2.50%, respectively) was added to the above solution with constant magnetic stirring for 2 h under a Xe lamp. Finally, after centrifugal separation, the obtained gray solid powder was washed with deionized water and ethanol, collected, and then dried in a 60 °C oven for 24 h. According to the different deposition amounts of Pt on SnS_2_, the synthesized samples are denoted as SnS_2_−1.0Pt, SnS_2_−1.5Pt, SnS_2_−2.0Pt, and SnS_2_−2.5Pt.

### 3.3. Characterization

This part can be seen in Supporting Information.

### 3.4. Evolution of Photocatalytic TC Degradation

Under the irradiation of a 300 W Xe lamp (HF−GHX−XE−300, Shanghai Hefan Instrument Co., Ltd., Shanghai, China), the photocatalytic reactions of the as-prepared samples were evaluated by photodegradation of TC aqueous solution (20 mg/L). The synthesized sample (20 mg) was dispersed into an aqueous TC solution (60 mL), and the suspension was stirred in a dark environment for 30 min to reach adsorption/desorption equilibrium before turning on the light. Subsequently, the photocatalytic suspension system was sampled (3 mL) at specific time intervals during the process of light illumination and then centrifugated to remove the photocatalyst (10,000 rpm/min, 2 min). Finally, the absorbance of the residual TC was measured using a UV−Vis spectrophotometer at 357 nm [42]. For comparison, the degradation of TC under light illumination without a photocatalyst, the degradation of TC with a photocatalyst under no light, and the degradation of TC in the absence of a photocatalyst and light were also investigated.

## 4. Conclusions

In summary, Pt NPs loaded on SnS_2_ sheet hybrids were successfully designed and synthesized via a simple hydrothermal approach and photodeposition process for photocatalytic TC degradation. Benefiting from the formation of a metal/semiconductor interface field between SnS_2_ and Pt enhancing the separation of photo-induced charge carriers, the LSPR effect of Pt strengthening the light absorption, and the sheet-like structure shortening the transfer path of charge carriers, the best catalyst displayed an excellent photocatalytic activity of TC degradation with a degradation efficiency of 91.27%, and a reaction rate constant of 0.0187 min^−1^, and durability. Our finding not only proposes a feasible strategy for utilizing the combined capabilities of sheet-like structures and the LSPR effect of Pt NPs but also paves a new avenue for the design of efficient and sustainable photodegradable materials for wastewater treatment technologies.

## Figures and Tables

**Table 1:** Comparison of TC photodegradation activity of previously published papers with SnS_2_-based catalysts.

Catalyst	Initial Concentration of TC (mg/L)	Dosage (g/L)	Reaction Time (min)	Degradation (%)	Kinetic Constant (min^−1^)	Ref.
**Pt/SnS_2_**	**20**	**0.33**	**120**	**91.27**	**0.0187**	**This work**
Fe/SnS_2_/Kaolinite ^a^	40	0.50	60	80.38	0.0257	48
Zn_2_SnO_4_/SnS_2_	10	0.10	120	83.00	0.0176	56
BiVO_4_/SnS_2_	10	0.20	150	80.80	0.0100	57
LaFeO_3_/SnS_2_	50	0.33	120	28.80	0.0028	58
Bi_2_MoO_6-x_/SnS_2_	20	0.20	90	89.00	0.0139	59
Ti_3_C_2_/SnS_2_	10	0.50	90	87.70	0.0156	60

^a^ A total of 8 mM of H_2_O_2_ was added to the reaction system.

## Data Availability

Data are contained within the article and Supplementary Materials.

## Supplementary Materials

The following supporting information can be downloaded at https://www.mdpi.com/article/10.3390/molecules29225423/s1: Figure S1: The energy-dispersive spectroscopy of SnS2−2.0Pt; Figure S2: The standard curve of absorbance vs. various concentrations of TC; Figure S3: (a) XRD patterns of fresh and used SnS2−2.0Pt and (b) a TEM image of used SnS2−2.0Pt; Figure S4: Photocatalytic TC degradation curves under various pH values of initial of TC; Table S1: A summary of peak area ration and the full width at half maximum of each peak for SnS2−2.0Pt according to XPS peak-fitting results.
