# Supplementary material for: Insight into the Local Surface Plasmon Resonance Effect of Pt-SnS2 Nanosheets in Tetracycline Photodegradation
Source: Molecules. 2024 Nov 17;29(22):5423. doi: 10.3390/molecules29225423 (PMC11597053; doi:10.3390/molecules29225423)
Supplement: Supplementary file 1 [file molecules-29-05423-s001.zip › molecules-3305464-supplementary.pdf]

Supporting Information

Insight into the Local Surface Plasmon Resonance Effect of Pt-SnS_2 Nanosheets in Tetracycline Photodegradation

Mao Feng [1], Tianhao Zhou [2], Jiaxin Li [2], Mengqing Cao [2], Jing Cheng [2], Danyang Li [2,3,*], Jian Qi [4,5,*] and Feifei You [2,*]

1 Textile School, Zhejiang Fashion Institute of Technology, Ningbo 315211, China; xgf@msn.com
2 College of Textile and Clothing, Yancheng Institute of Technology, Yancheng 224051, China; 17311504196@163.com (T.Z.); 19705102579@163.com (J.L.); 18164327219@163.com (M.C.); 19705100129@163.com (J.C.)
3 Sichuan Provincial Engineering Research Center of Functional Development and Application of High Performance Special Textile Materials, Chengdu Textile College, Chengdu 611731, China
4 State Key Laboratory of Biochemical Engineering, Institute of Process Engineering, Chinese Academy of Sciences, Beijing 100190, China
5 School of Chemical Engineering, University of Chinese Academy of Sciences, Beijing 100049, China
* Correspondence: strawberry4173@163.com (D.L.); jqi@ipe.ac.cn (J.Q.); ffyouycit@163.com (F.Y.)

.

Synthesis

Preparation of carbonaceous microspheres (CMSs) templates: CMSs templates were fabricated with the hydrothermal polymerization reaction of sucrose. Briefly, 130 g of sucrose matching with 250 mL of deionized (DI) water stirred vigorously under a constant magnetic stirring until it thoroughly dissolved in a beaker at room temperature. Next, the well-mixed solution was transferred into a 500 mL of Teflon-lined autoclave, sealed and put them into a preheating oven at 200 ºC for 123 min. After the autoclave cooling to room temperature naturally, the resulting dark brown precipitates were gathered via vacuum filtration method, washing with DI water for three times. Finally, the dark brown precipitates were heated in oven at 60 ºC for overnight.

Preparation of SnO_2 hollow structured microspheres: SnO_2 hollow microspheres were prepared by hard template method. In short, 1 g of CMSs was soaked into 0.2 M $SnCl_4$ aqueous and alcohol mixed solution (40 mL, volume ratio of deionized water to ethanol was 1:1) under magnetic stirring for 4 hours at room temperature. And then, the black precipitates were collected through filtration, washed with DI water for three times and dried at 70 ºC for 12 h. Next, the drying black precipitates were fully ground and laid flat in the porcelain boat. And the grinding solid were further annealed with a procedure heating rate of 2 ºC /min and held at 500 °C for 120 min in air environment muffle furnace so as to eliminate the CMSs templates.

Characterization

The crystal phase structures of synthetic photocatalysts were explored by Powder X-ray diffraction (XRD), which observed on a Panalytical X'Pert PRO MPD [Cu Ka radiation (λ, 1.505 Å)] with the operating voltage of 40 kV and current of 30 mA. Transmission electron microscopy (TEM) and high-resolution TEM (HRTEM) images were analyzed on a FEI Tecnai F20 instrument with an accelerating voltage of 200 kV. High-angle annular dark field-scanning transmission electron microscopy (HAADF-STEM) and elemental mapping images, which were used to ensure the

catalysts element distribution, were observed by Tecnai G2 F20 U-TWIN. X-ray photoelectron spectroscopy (XPS) was measured to study the surface chemical states and elements electronic structures of samples, including Sn, S and Pt, using an ESCALab220i-XL electron spectrometer (VG Scientific) with Al Kα radiation of 300 W. The UV-Vis spectrometer (Cary 5000, Varian) integrated with an integrating sphere was employed to capture the bandgaps of the as-synthesized samples, where $BaSO_4$ was used as the reference. The steady-state and time-resolved photoluminescence (PL) spectra were probed via a fluorescence spectrophotometer (Cary Eclipse) with an excitation wavelength of 340 nm at room temperature. Photoelectrochemical properties including, electrochemical impedance spectroscopy (EIS), Mott-Schottky plots and time-resolved photocurrent behaviors were carried out on the electrochemical workstation (CH Instruments Ins.) with a three-electrode system with the integrated photoelectrodes. And SnS_2-Pt samples (put on FTO glass, 1.0×1.0 cm^2), Pt slice electrode and Ag/AgCl electrode were used as the working electrode, counter electrode and reference electrode, respectively. 0.1M Na_2SO_4 solution was also utilized as electrolyte.

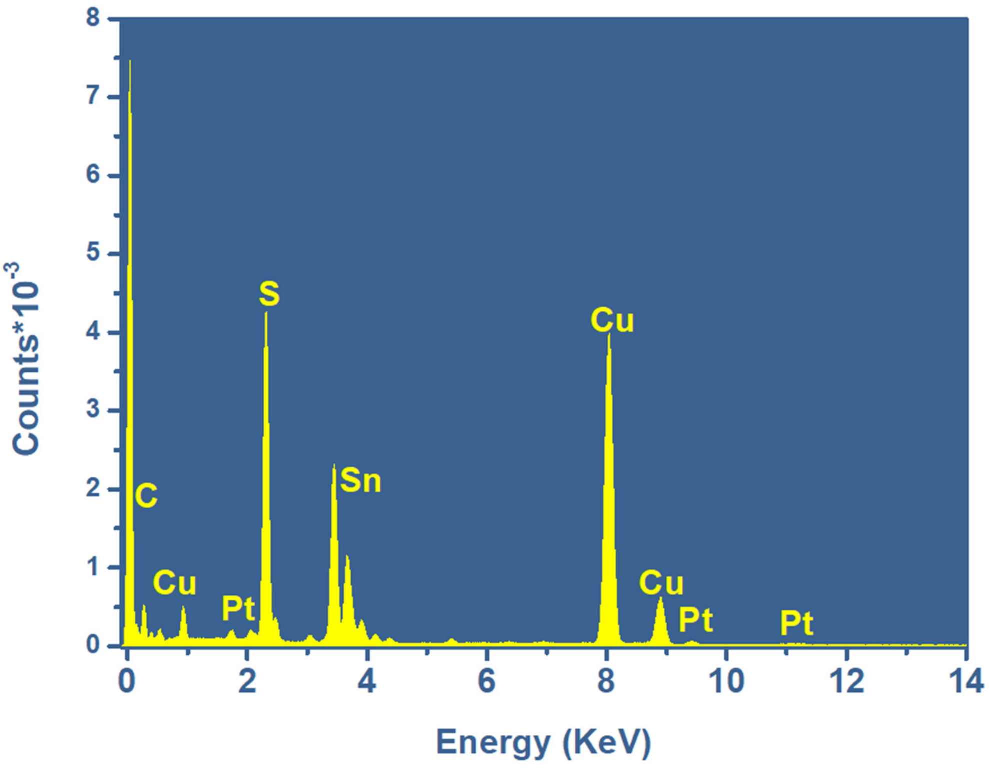

Figure S1. The energy dispersive spectroscopy of SnS_2-2.0Pt (The detected elements of C and Cu was caused by the carbon support network, which was the carrier of sample during characterization).

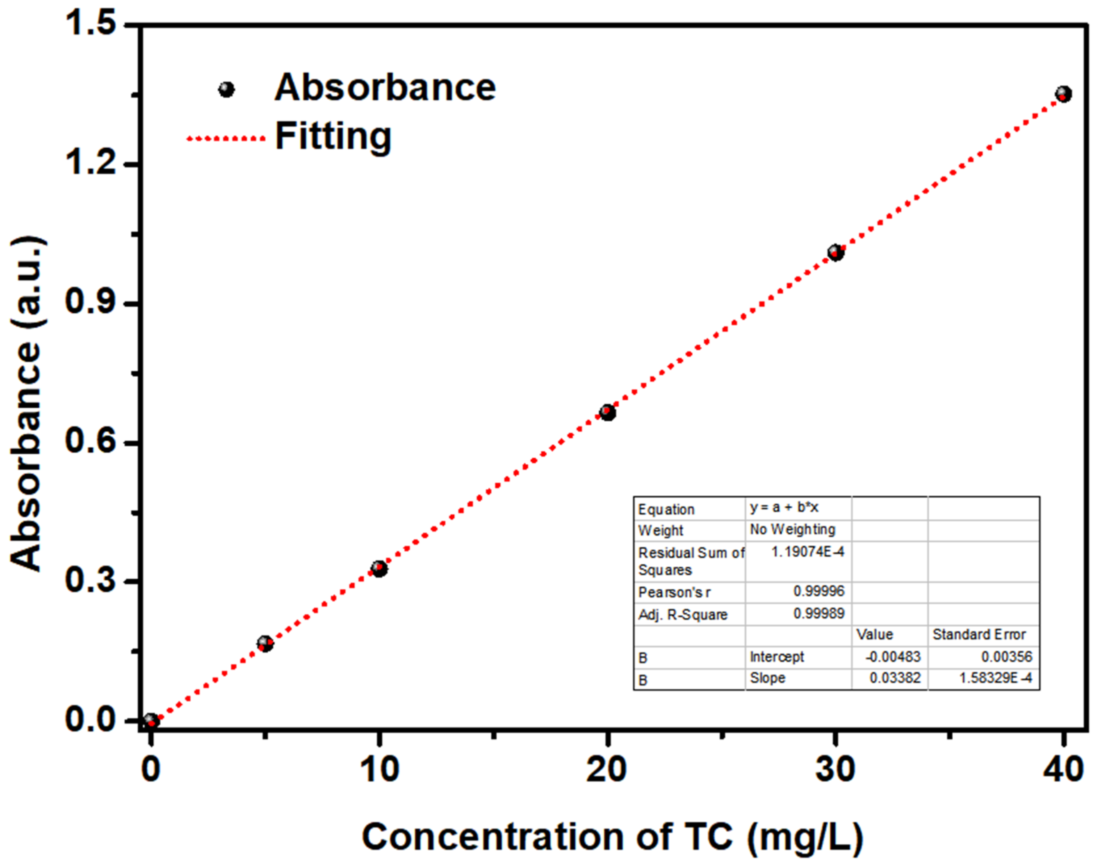

Figure S2. The standard curve of absorbance *vs.* varies concentration of TC.

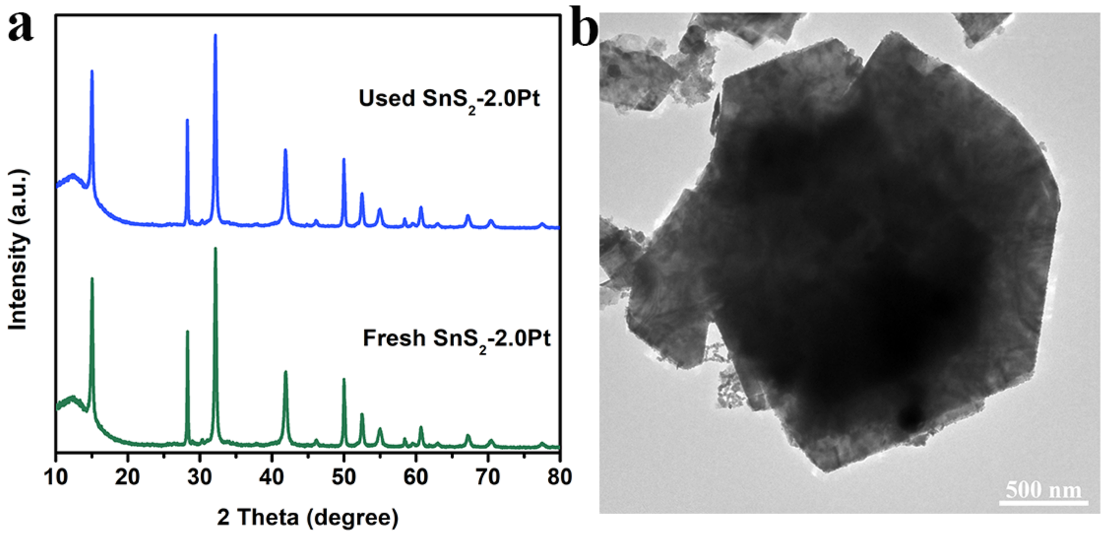

Figure S3. (a) XRD patterns of fresh and used SnS_2-2.0Pt and (b) TEM image of used SnS_2-2.0Pt.

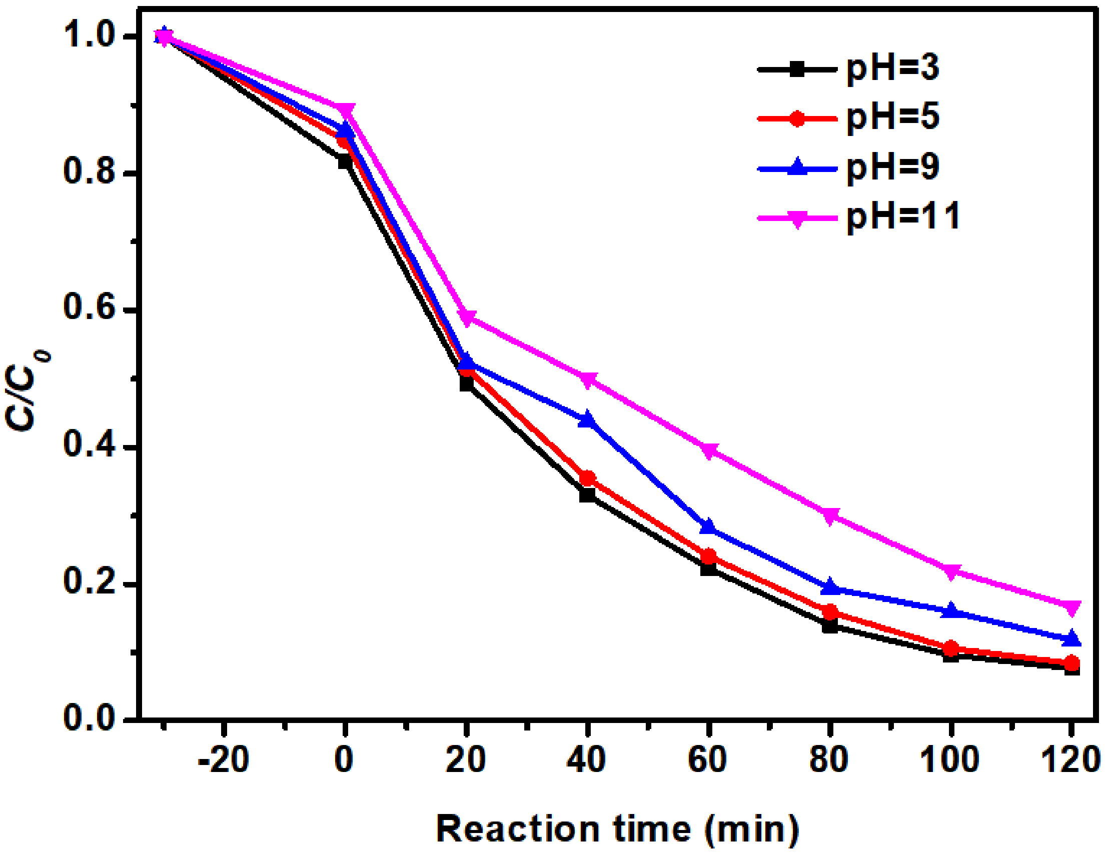

Figure S4. Photocatalytic TC degradation curves under varies pH vales of initial of TC.

Table S1. A summary of peak area ration and the full width at half maximum of each peak for SnS_2-2.0Pt according to XPS peaks fitting results.

Species	Peak position (eV)	FWHM[a] (eV)	Area	Ratio[b] (%)
Pt^0	75.1	1.22	33798	70.18
	71.7	1.16	35606	
Pt^{2+}	76.3	1.38	12863	29.82
	73.0	1.33	16631	

[a] FWHM stands for the full width at half maximum. [b] The ratios are calculated by dividing the area of each peak by the total area.
